# Safety and efficacy of Intacs in Indian eyes with keratoconus: An initial report

**DOI:** 10.4103/0301-4738.77036

**Published:** 2011

**Authors:** Anand Parthasarathy

**Affiliations:** Head, Cornea Services, Vasan Eye Care Hospitals, Chennai, India

Dear Editor,

I read with interest the article by Shetty *et al*.[1] on the safety and efficacy of Intacs. The authors need to be commended on reporting the results of the procedure they carried out on Indian eyes. The authors would, however, do well to shed some light on some aspects of their results; the mean refractive spherical equivalent (MRSE) drop from -5.48 ± 3.67 to -1.43 ± 2.74 and then an increase to -5.23 ± 3.79 again is surprising. Why did the variation take place and is there a possible explanation for this? Second, in the preoperative examination, was the presence of vernal keratoconjunctivitis specifically looked for, as we know that there is an association between the two;[2] in addition postoperatively these patients need to be counseled about avoiding rubbing their eyes, as it could lead to progression of keratoconus and even extrusion of the implant. Also how many of the patients in the case series had collagen cross-linking prior to the Intacs placements? There are now multiple studies confirming a possible synergistic effect of the two treatments.[3–5]
